# Endocytosis, intracellular fate, accumulation, and agglomeration of titanium dioxide (TiO_2_) nanoparticles in the rainbow trout liver cell line RTL-W1

**DOI:** 10.1007/s11356-019-04856-1

**Published:** 2019-03-31

**Authors:** Tobias Lammel, Aiga Mackevica, Bengt R. Johansson, Joachim Sturve

**Affiliations:** 1 Department of Biological and Environmental Sciences, University of Gothenburg, Box 463, 413 90 Göteborg, Sweden; 2 DTU Environment, Technical University of Denmark, 2800 Kongens Lyngby, Denmark; 3 The Electron Microscopy Unit, Institute of Biomedicine, Sahlgrenska Academy at University of Gothenburg, 405 30 Göteborg, Sweden

**Keywords:** Endocytosis, Particokinetics, Bioaccumulation, Hepatocyte, Fish

## Abstract

There is increasing evidence that titanium dioxide (TiO_2_) nanoparticles (NPs) present in water or diet can be taken up by fish and accumulate in internal organs including the liver. However, their further fate in the organ is unknown. This study provides new insights into the interaction, uptake mechanism, intracellular trafficking, and fate of TiO_2_ NPs (Aeroxide® P25) in fish liver parenchymal cells (RTL-W1) in vitro using high-resolution transmission electron microscopy (TEM) and single particle inductively coupled plasma mass spectrometry (spICP-MS) as complementary analytical techniques. The results demonstrate that following their uptake via caveolae-mediated endocytosis, TiO_2_ NPs were trafficked through different intracellular compartments including early endosomes, multivesicular bodies, and late endosomes/endo-lysosomes, and eventually concentrated inside multilamellar vesicles. TEM and spICP-MS results provide evidence that uptake was nano-specific. Only NPs/NP agglomerates of a specific size range (~ 30–100 nm) were endocytosed; larger agglomerates were excluded from uptake and remained located in the extracellular space/exposure medium. NP number and mass inside cells increased linearly with time and was associated with an increase in particle diameter suggesting intracellular agglomeration/aggregation. No alterations in the expression of genes regulated by the redox balance-sensitive transcription factor Nrf-2 including superoxide dismutase, glutamyl cysteine ligase, glutathione synthetase, glutathione peroxidase, and glutathione S-transferase were observed. This shows that, despite the high intracellular NP burden (~ 3.9 × 10^2^ ng Ti/mg protein after 24 h) and NP-interaction with mitochondria, cellular redox homeostasis was not significantly affected. This study contributes to a better mechanistic understanding of in vitro particokinetics as well as the potential fate and effects of TiO_2_ NPs in fish liver cells.

**Electronic supplementary material:**

The online version of this article (10.1007/s11356-019-04856-1) contains supplementary material, which is available to authorized users.

## Introduction

TiO_2_ NPs are emerging environmental contaminants to which aquatic biota including fish will become increasingly exposed (Johnson et al. 2011; Kaegi et al. 2008; Kiser et al. 2009; Mueller and Nowack 2008; Peters et al. 2018; Weir et al. 2012). There is increasing evidence that water- and diet-borne exposure results in TiO_2_ NP uptake, systemic distribution, and accumulation in internal organs including the liver (Al-Jubory and Handy 2013; Federici et al. 2007; Ramsden et al. 2009). For instance, Ramsden et al. (2009) measured a significant increase in hepatic Ti concentration (approximately 0.05–0.1 mg Ti g^−1^ dry weight tissue) in rainbow trout following dietary exposure to TiO_2_ NPs (10 mg TiO_2_ kg^−1^ food). Also, their results showed limited elimination of Ti from the liver after 2 weeks of depuration (Ramsden et al. 2009). Furthermore, we recently observed a TiO_2_ NP-resembling electron-dense object in the perisinusoidal space of brown trout liver fed TiO_2_ NP-containing diet suggesting that TiO_2_ NPs can cross the sinusoidal endothelium and come in direct contact with liver parenchymal cells (manuscript under review (Lammel et al. 2019)). These observations raise several questions: Are TiO_2_ NPs that reach the liver endocytosed by liver parenchymal cells? Which is the size spectrum of TiO_2_ NPs that can be taken up? Through which endocytic mechanism are they taken up? Which is their further fate and destination inside the cell? Can they be eliminated or will they accumulate with time? And, can accumulation result in adverse effects?

In vitro experimental systems based on cultured cells provide a versatile toxicological tool that can be used to study cellular uptake and toxicity of NPs and aid in answering these questions—in combination with appropriate analytical techniques (Chithrani et al. 2006; dos Santos et al. 2011; Iversen et al. 2011; Lammel and Navas 2014). However, various aspects need to be considered when studying NP uptake and toxicity in vitro as well as when interpreting the obtained data. NPs in medium dispersions used for cell exposure are seldom present as single NPs, but as agglomerates or aggregates of various sizes and different effective densities—even if protocols are optimized for NP stability (Casanova et al. 2011; Ji et al. 2010; Lammel and Sturve 2018; Taurozzi et al. 2013). The behavior and fate of different-sized and dense agglomerates in the in vitro system are likely to be different. For instance, larger agglomerates may settle faster to the bottom of the culture vessel than smaller agglomerates, and very small agglomerates and single NPs may remain colloidally dispersed. This differential behavior and fate can result in complex and dynamic exposure conditions, which are challenging to characterize and monitor (Cohen et al. 2015; Cohen et al. 2014; DeLoid et al. 2015). Besides, one needs to bear in mind that complexity further increases when NPs/agglomerates interact with the cell surface (Nel et al. 2009). Different particle sub-populations co-existing within the exposure medium may differ in their properties, not only in their size and shape but also their biocorona composition altering surface chemistry and charge (Hu et al. 2014; Lundqvist et al. 2008; Piella et al. 2017). These properties are known to determine the type and strength of interactions with plasma membrane proteins and lipids, which in turn will decide if, via which mechanism and with which relative efficiency particles are taken up (Frohlich 2012; Jiang et al. 2015; Nel et al. 2009; Oh and Park 2014; Reifarth et al. 2018; Zhang et al. 2015; Zhao et al. 2011). NPs can enter cells via different routes including clathrin-mediated endocytosis, caveolin-mediated endocytosis, micropinocytosis, phagocytosis as well as by spontaneous translocation across the plasma membrane (Oh and Park 2014; Zhao et al. 2011). The uptake pathway determines the further intracellular fate and final destination of the particles in the cells and hence is likely to influence their toxicity. Thus, selective uptake of particle sub-populations leads to a situation, where the cells are exposed to different particle sub-populations from outside the cell than from inside the cell. Unraveling to which extent the different particle sub-population contributes to observed toxic effects is challenging and requires temporal and spatial information on their respective fate and relative distribution in the in vitro system. Thus, characterization of the NPs’ behavior and fate in the cell culture medium, such as hydrodynamic size distribution and stability, may not suffice when one wants to fully understand NP toxicity, but additional, ideally both imaging and quantitative data on the internalized NPs including uptake pathway, uptake rate, intracellular fate, and accumulation may be needed.

There is previous evidence that NPs can be taken up by different types of fish cells (Felix et al. 2017; Gaiser et al. 2012; Kalman et al. 2019; Kuhnel et al. 2009; Lammel and Navas 2014; Picchietti et al. 2017; Scown et al. 2010; Van Hoecke et al. 2013; Yue et al. 2016). Furthermore, for some NPs, valuable information on the uptake pathway and intracellular distribution could be obtained by different analytical techniques, such as laser scanning confocal fluorescence microscopy (Felix et al. 2017; Gaiser et al. 2012; Scown et al. 2010; Van Hoecke et al. 2013), flow cytometry (Felix et al. 2017), ICP-MS analysis of subcellular fractions (Yue et al. 2016), as well as scanning and transmission electron microscopy (Kalman et al. 2019; Kuhnel et al. 2009; Lammel and Navas 2014; Picchietti et al. 2017; Van Hoecke et al. 2013; Yue et al. 2016). However, to the best of our knowledge, the scientific literature does not seem to comprise any comprehensive study that investigated cellular uptake, accumulation, and intracellular fate of TiO_2_ NP in a piscine liver cell model—despite the fact that the liver is a likely target organ (Al-Jubory and Handy 2013; Federici et al. 2007; Ramsden et al. 2009).

The objective of this study was to fill this gap of knowledge. Specifically, we aimed at (1) identifying the endocytic uptake pathway, (2) determining the endocytosable size range, (3) getting insight into the further intracellular transport route and fate, and (4) quantifying the increase in intracellular dose in the rainbow trout liver cell line RTL-W1. This cell line is an appropriate model in this context because it is derived from the same species for which hepatic TiO_2_ NP accumulation was reported in vivo (Al-Jubory and Handy 2013; Federici et al. 2007). Furthermore, it has spontaneously arisen from normal liver tissue without having undergone neoplastic transformation (Lee et al. 1993), which makes it likely that its endocytic machinery and endo-lysosomal functionalities resemble that of hepatocytes in vivo. In addition, it has established itself as an important in vitro model in aquatic toxicology and is increasingly used to study NP toxicity and underlying mechanisms (Bermejo-Nogales et al. 2017; Connolly et al. 2015; Fernandez et al. 2013; Galbis-Martinez et al. 2018; Simon et al. 2014; Vo et al. 2014). Therefore, knowledge on mechanisms of NP uptake and potential intracellular transport routes in this cell line is of high interest, as it will benefit future studies in this area including mechanistic effect studies, chronic toxicity studies, and mixture toxicity studies.

Recently, we have established a dosing procedure based on work by Taurozzi et al. (2013), which allows exposure of RTL-W1 cells to TiO_2_ NPs under highly controlled conditions, where the NPs are maximally and stably dispersed and hydrodynamic size distribution does not change with time and exposure concentration (Lammel and Sturve 2018). In the present study, we make use of this fully characterized system to advance our understanding of the uptake and fate of TiO_2_ NPs in fish liver cells using two analytical techniques, transmission electron microscopy (TEM) and single particle inductively coupled plasma mass spectrometry (spICP-MS), which can provide complementary, that is, qualitative and quantitative information on metal NP content and size inside cells at high-resolution (Deng et al. 2017; Montano et al. 2016; Reifarth et al. 2018). Moreover, we use reverse transcriptase-quantitative polymerase chain reaction (RT-qPCR) to measure expression levels of genes regulated by the redox-sensitive transcription factor Nrf-2 in order to determine whether TiO_2_ NPs do perturb the cellular redox homeostasis at the described exposure conditions.

## Material and methods

### Chemicals

Bovine serum albumin (BSA), ethylenediaminetetraacetic acid (EDTA), poly-L-lysine solution poly-L-lysine (MW 70,000-150,000, 0.01%, sterile-filtered), formaldehyde solution, ethanol, acetone, fluorescamine, and nystatin (bioreagent, suitable for cell culture) were purchased from Sigma-Aldrich Sweden AB (Stockholm, SE).

### Nanomaterial

Titanium dioxide (TiO_2_) nanopowder (primary particle size (TEM) 21 nm; surface area (BET) 35–65 m^2^/g; purity ≥ 99.5% trace metals basis) was purchased from Sigma-Aldrich (product number 718467; synonym: Aeroxide® P25, Titania, Titanium (IV) oxide).

### Preparation of TiO_2_ NP stock dispersions

TiO_2_ NP stock dispersions were prepared as described in Lammel and Sturve (2018). In brief, 100 mg of TiO_2_ nanopowder was weighed into 15-ml polypropylene tubes (Sarstedt, Nümbrecht, DE) and dispersed in 10 ml Milli-Q water (18.2 MΩ cm at 25 °C) using a Branson 250 sonifier equipped with a 3-mm-diameter tapered microtip (Branson Ultrasonics Corporation, Danbury, CT, USA) operated at 10% maximum amplitude (corresponding to ~ 20 W) in pulse mode (1 s on/1 s off). The total sonication time was 30 min (ultrasound was applied for 15 min). To prevent heating during ultrasonic dispersion, the 15-ml processing tube was immersed in an ice-water bath (0 °C). The resulting 10 mg/ml TiO_2_ NP/Milli-Q dispersions were autoclaved and stored as stock dispersions at 4 °C in the dark (Lammel and Sturve 2018).

### Preparation of TiO_2_ NP dispersions in cell culture medium

TiO_2_ NP dispersions in cell culture medium were prepared as previously described (Lammel and Sturve 2018). In brief, autoclaved 10 mg/ml TiO_2_ NP stock dispersions were diluted in sterile Milli-Q water to obtain intermediate stock dispersions that were of 100-times higher concentration than the targeted NP concentration in Leibovitz’s L-15 medium (Gibco, Thermo Fisher Scientific). The intermediate stock dispersions (concentrations 10, 1, and 0.1 mg/ml) were then diluted 1:10 in sterile-filtered (0.2 μm filtropur syringe filter; Sarstedt) aqueous 10 mg/ml BSA solution, and the resulting TiO_2_ NP/BSA dispersions (concentrations 1, 0.1, and 0.01 mg/ml) were further diluted 1:10 in serum-free L-15 medium yielding BSA-stabilized TiO_2_ NP dispersions with concentrations of 100, 10, and 1 μg/ml, respectively. These dispersions were used for cell exposures (see below).

### Dynamic light scattering analysis

Dynamic light scattering (DLS) analysis was performed on a Zetasizer Nano-ZS apparatus (Malvern Instruments Ltd., Malvern, UK) using disposable polystyrene macro cuvettes (VWR International AB, Göteborg, SE). Of each type of dispersion (stock dispersions with appropriate concentrations 1 and 0.1 mg/ml; TiO_2_ NP/BSA dispersions, concentrations 1, 0.1, and 0.01 mg/ml; TiO_2_ NP culture medium dispersions, concentrations 100, 10, and 1 μg/ml), three independent samples prepared from different TiO_2_ NP stock dispersions were measured. For each sample, four consecutive measurements at ten runs were conducted using 173° backscatter detection. The instrument automatically determined the attenuation level and optimum measurement position. The measurement temperature was set to 20 °C, which corresponds to the temperature in cell culture experiments. The general purpose (normal resolution) analysis model was selected for result calculation. The software used for analysis and visualization of DLS data was the Zetasizer software version 7.11 (Malvern Instruments Ltd.).

### Zeta-potential determination

Zeta-potential was determined in diluted TiO_2_ NP stock dispersions and TiO_2_ NP/BSA dispersions (100 and 10 μg/ml) measuring electrophoretic mobility of the particles at 20 °C via laser Doppler velocimetry using a Zetasizer Nano-ZS apparatus (Malvern Instruments Ltd.) and disposable capillary cuvettes (Malvern Instruments Ltd.). Three consecutive measurements were taken of each sample. The number of runs was set automatically. The Smoluchowski approximation was used for calculation.

### Cell culture

RTL-W1 cells were cultured in 75 cm^2^ cell culture flasks (TC Flask T75, Cell+, Sarstedt) in phenol red-free Leibovitz’s L-15 Medium (Gibco) supplemented with 5% fetal bovine serum (FBS) (Gibco). The flasks were incubated at 19 °C and split in ratios of 1:2 or 1:3 when reaching confluence using 0.2 g/L ethylenediaminetetraacetic acid (EDTA)/phosphate-buffered saline (PBS) and 0.25% trypsin-EDTA solution (Gibco).

### Study of TiO_2_ NP uptake and intracellular fate using transmission electron microscopy

#### Seeding

RTL-W1 cells were seeded onto sterilized (EtOH + 1 h UV) Thermanox™ coverslips (Ø 13 mm) placed in the wells of a 24-well plate (Sarstedt). The cell density at the time of seeding was 12 × 10^4^ cells/well, or ~ 6.6 × 10^4^ cells/cm^2^. The volume of culture medium (L-15 containing 5% FBS) per well was 1 ml.

#### Exposure

After 24 h of incubation at 19 °C, the cells were washed with serum-free L-15 medium and exposed to 100 μg/ml TiO_2_ NP. Besides, a negative control was included. The negative control consisted of cell cultures treated with NP-free culture medium. After 15 min, 30 min, 2.5 h, 4 h, and 24 h of incubation, the exposure medium was removed and the cells carefully rinsed with PBS containing Ca^2+/^Mg^2+^. Thereafter, the cells were fixed in 2% formaldehyde + 2.5% glutaraldehyde + 0.02% Na azide in 0.05 M Na cacodylate diluted 1:1 with PBS for 1 h at 4 °C.

#### Sample preparation and analysis

Cells were postfixed with 1% osmium tetroxide (OsO_4_) and 1% potassium ferrocyanide for 2 h at 4 °C. Cells were dehydrated with increasing concentrations of ethanol ending in acetone and embedded in Agar 100 resin (Agar Scientific Ltd., UK). Resin-embedded cell cultures were cut perpendicular to the plane of the Thermanox™ surface using a Leica UC60 ultramicrotome. The obtained ultrathin sections (~ 60 nm) were collected on Formvar-coated grids and counterstained with 1% uranyl acetate and Reynolds’ lead citrate (Agar Scientific Ltd., UK) and then examined using a Zeiss Leo 912 AB transmission electron microscope (Zeiss, Germany) operated at 120 kV. Digital images were recorded with a Veleta CCD camera. The ESIvision software was used for image capture and processing.

### Energy dispersive X-ray analysis

Energy dispersive X-ray (EDX) analysis was performed using an FEI Tecnai T20 and an EDAX detector. Data were processed using the TEM Imaging and Analysis (TIA) software.

### Imaging of intracellular TiO_2_ NPs using confocal laser scanning microscopy

#### Seeding

RTL-W1 cells were seeded onto poly-L-lysine-coated glass coverslips (Ø 13 mm) placed in the wells of a 24-well plate (Sarstedt). The cell density at the time of seeding was 10 × 10^4^ cells/well, or ~ 5.5 × 10^4^ cells/cm^2^. The volume of culture medium (L-15 containing 5% FBS) per well was 1 ml.

#### Exposure

After 24 h of incubation at 19 °C, the cells were washed with serum-free L-15 medium and exposed to 100 μg/ml TiO_2_ NP. The cells were carefully rinsed with L-15/ex and fixed in 4% paraformaldehyde in PBS for 20 min at room temperature. L-15/ex is a minimal version of L-15 medium used here as a physiological buffer solution instead of PBS containing Ca^2+/^Mg^2+^ for cell washing (Schirmer et al. 1997).

#### Sample preparation and analysis

The coverslips were mounted on a microscope slide for confocal laser scanning microscopy (CLSM) analysis using ProLong® Gold Antifade Mountant containing the nuclear stain DAPI (Thermo Fisher Scientific). Cells were imaged using a Zeiss LSM 710 NLO system. TiO_2_ NPs were visualized in reflection mode. CLSM images were analyzed using the ZEN 2.1 SP1 (black) software from Zeiss.

### Determination of intracellular particle size distribution and mass concentration using single-particle inductively coupled plasma mass spectrometry

#### Seeding

RTL-W1 cells were seeded into 60 × 15 mm cell culture dishes (Sarstedt) by adding 6 ml of a single-cell suspension containing 0.3 × 10^6^ cells/ml and subsequently incubated for 24 h at 19 °C to allow attachment and formation of a confluent monolayer (growth area normalized cell density 8.5 × 10^4^ cells/cm^2^).

#### Exposure

Thereafter, cells were pre-incubated with 50 μM nystatin or only medium (control) for 90 min. The nystatin concentration was chosen based on literature showing its effectiveness to inhibit caveolae-mediated endocytosis and the absence of effects on cell viability (Ivanov 2008). In parallel, cells were incubated in medium without TiO_2_ NP or nystatin as a negative control. At the end of the exposure, cells were washed twice with DPBS (5 ml) to remove medium, and any NP residues washed again twice with PBS/EDTA (5 ml) and then trypsinized and collected in L-15 containing 5% FBS (10 ml). Note that successful removal of cell surface-adsorbed NPs was verified by photospectrometric measurements as previously described (Lammel and Sturve 2018). The obtained cell suspensions were centrifuged for 5 min at 1500 rpm, the supernatant discarded, and the pellet resuspended in DPBS (5 ml). After that, cells were pelleted a second time, lysed by adding 1 ml Milli-Q water (after removal of the supernatant), homogenized, and stored frozen at − 20 °C until used for Ti analysis by spICP-MS. Before freezing, an aliquot was removed for total protein content determination using the fluorescamine assay. In brief, following 1:4 dilution in Milli-Q water, the aliquots were added to a 96-well (100 μl/well). In parallel, a dilution series of a BSA standard was prepared in Milli-Q water and added to the same plate (concentration range 1–1000 μg/ml). Thereafter, 50 μl of 0.3 mg/ml fluorescamine solution in acetonitrile was added to each well. The plate was incubated on a shaker in the dark for 10 min, and then the fluorescence intensity was measured at 360 nm excitation/450 nm emission using a Wallac 1420 Victor multilabel counter (Wallac Oy, Turku, Finland). The calculated protein concentrations were used for normalization of intracellular Ti contents determined by spICP-MS.

#### Sample preparation and analysis

spICP-MS analysis was performed to measure TiO_2_ particle size distribution and particle number concentration. Samples were analyzed for ^48^Ti isotope, assuming particle density of 3.9 g/cm^−3^, dwell time was set at 100 μs, measurement time 100 s, and the particle size was calculated based on the dissolved Ti calibration curve, which was prepared in 0.1% HNO_3_, and the transport efficiency was calculated using 60 nm Au particles (Perkin Elmer, USA). Cell lysate samples were diluted in Milli-Q water by a factor of 1000 to reach optimal particle number concentration for spICP-MS analysis (PerkinElmer, NexION 350D). The LOD (size) was 32 nm for TiO_2_, assuming spherical shape of the particles, and was calculated based on mean particle size for blank samples (pure Milli-Q water) and adding three standard deviations (*n* = 3). Data processing was done using the Syngistix software (v.2.1, Perkin Elmer).

### Gene expression analysis

The effect of TiO_2_ NP exposure on Nrf2 pathway activation was studied by RT-qPCR analysis. Confluent RTL-W1 cell cultures (7.5 × 10^5^ cells/well; seeded 24 h prior to treatment into Greiner CELLSTAR 6-well plates; growth area normalized cell density ~ 7.8 × 10^4^ cells/cm^2^) were exposed to 1, 10, and 100 μg/ml TiO_2_ NP dispersions for 24 h. Cells exposed to cell culture medium alone (including the corresponding amount of Milli-Q water and BSA) served as no treatment control. After exposure, cells were collected and the total RNA isolated using the RNeasy Plus Mini Kit (Qiagen) following manufacturer recommendations. In brief, cells were lysed using RLT Plus buffer including *β*-mercaptoethanol and QIAshredder spin columns, genomic DNA was removed using gDNA Eliminator spin columns, and total RNA was isolated and purified using RNeasy spin columns in combination with appropriate buffer solutions provided by the kit. The purified RNA was transcribed into cDNA using the iScript™ cDNA synthesis kit from Bio-Rad. The RNA concentration in the cDNA synthesis reactions was 25 ng/μl. Quantification of relative mRNA expression levels (cDNA copy numbers) of selected reference and oxidative stress-related target genes was carried out using SsoAdvanced™ Universal SYBR Green Supermix from Bio-Rad and appropriate primer pairs synthesized by Eurofins Genomics (Ebersberg, DE). The sequences of the primers used for ubiquitin (Ubq), Nrf-2, cytosolic superoxide dismutase (SOD-1), glutamate cysteine ligase catalytic subunit (GCLcat), glutathione synthetase (GS), glutathione peroxidase (GPx), and glutathione S-transferase (GST) are shown together with their concentration in the assay and literature reference in Table 1. The efficiency of the primers was 90–110%. No-reverse transcriptase controls and no-template controls were run in parallel. The concentration of the cDNA template in the reactions was 2.5 ng/μl. The volume of the reactions was 10 μl. All qPCR reactions were run on a Bio-Rad CFX Connect™ Real-Time System programmed to conduct one 3-min cycle at 95 °C followed by 40 cycles of 10 s at 95 °C (denaturation) and 30 s at 60 °C (annealing/extension). All reactions were run in duplicate. The entire experiment (TiO_2_ NP exposure) was repeated three times (*n* = 3).

**Table 1:** Primers and qPCR assay conditions

Gene name and NCBI reference sequence	Sequence (5′->’)		Conc (nM)	Temperature (°C)	Ref.
Ubiquitin (Ubq) (XM_021603493.1)	FW	ACAACATCCAGAAAGAGTCCA	300	60	a
	RV	AGGCGAGCGTAGCACTTG			
Nuclear factor erythroid 2-related factor 2 (Nrf-2) (XM_021597223.1)	FW	TTTGTCCCTTCCTGAGCTGC	500	60	b
	RV	GGGCAATGGGTAGAAGCTGT			
Cytosolic superoxide dismutase (SOD-1) (NM_001124329.01)	FW	CCAATCAGCTTCACAGGACCAT	300	60	c
	RV	AGGCTGTTTGCGTGCTCAA			
Glutathione cysteine ligase, catalytic subunit (GCLcat) (XM_021576493.1)	FW	TGAGGGAGTTTGTGGACAAAGC	300	60	a
	RV	AATAGTTCTGGCATCGCTCCTC			
Glutathione synthetase (GS) (XM_021616063.1)	FW	TGGCTTTGGCAAAACCAGACC	500	60	a
	RV	GCCATCCTCTCTGTGCTCTGC			
Glutathione peroxidase (GPx) (XM_021585624.1)	FW	CCTGGGAAATGGCATCAAAGT	300	60	c
	RV	GGGATCATCCATTGGTCCATAT			
Glutathione S-transferase (GST) (XM_021561454.1)	FW	ACCTGGTGCTCTGCTCCAGTT	300	60	c
	RV	AGAGCTCAGGAAGCCCTTGAT			

^a^Carney Almroth et al. 2008

^b^Birgersson 2015

^c^Gunnarsson et al. 2009

Relative gene expression levels were calculated using the 2^-ΔΔCt^ method (Livak and Schmittgen 2001). ΔCq values were calculated subtracting the mean Cq value of each target gene by the mean Cq value of the reference gene Ubq, which was stably expressed. The fold change (FC) in the expression level of the target genes in each treatment compared to the no treatment control was calculated as FC = 2^-ΔΔCq^, whereas ΔΔCq was calculated by subtracting the ΔCq values obtained for the no treatment control from the ΔCq values obtained for the treatments. Statistical comparisons of gene expression levels between treatments were carried out on ΔCq values by one-way repeated measures analysis of variance (one-way RM ANOVA). All data were tested for normal distribution and homoscedasticity using a Shapiro-Wilk Normality and Equal Variance Test. The statistics software used was the SigmaPlot for Windows Version 12.0 (Systat Software, Inc.).

## Results

### Characterization of exposure conditions

As outlined in the introduction, a thorough characterization of the NPs’ properties, behavior, and fate in the exposure medium is essential when studying NP uptake and toxicity in vitro. In this study, we followed a previously established dosing procedure that results in maximally disaggregated and stable TiO_2_ NP dispersions allowing highly controlled cell exposures (Lammel and Sturve 2018). Comprehensive data on the morphology and size distribution of the TiO_2_ NPs in the commercial nanopowder, their hydrodynamic size-intensity distribution and colloidal stability in Milli-Q water (=stock dispersion), BSA solution (=intermediate working dispersion), and serum-free L-15 culture medium (=dispersion used for exposure) are available and discussed in Lammel and Sturve (2018).

**Fig. 1:**
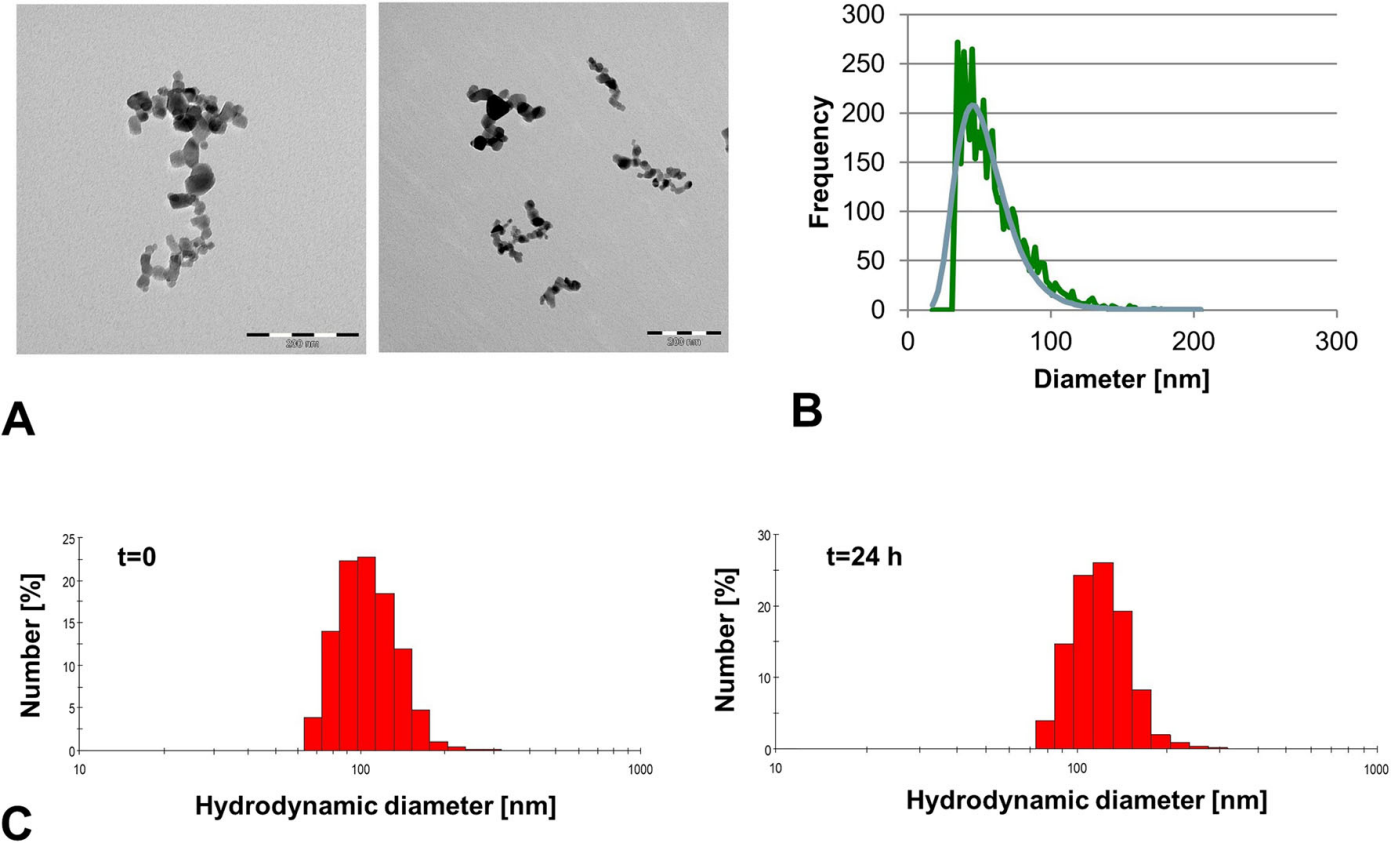
Shape and size of TiO2 NPs. **a** TEM images showing particle size and shape of pristine TiO_2_ NPs. Scale bars in the left and right image correspond to 200 nm. **b** Particle size-frequency distribution in TiO_2_ NP stock dispersion measured by spICP-MS. **c** Hydrodynamic size-frequency distribution by number in L-15 cell culture medium measured by DLS at *t* = 0 and after 24 h of incubation (left and right images, respectively)

The primary particle shape and mean size of individual TiO_2_ Aeroxide® P25 NPs corresponded to that reported by the manufacturer (near-spherical, 21 nm in diameter) (Fig. 1a) (Lammel and Sturve 2018). Smaller (down to 10 nm) and larger (up to ~ 100 nm) individual particles were observed as well (Lammel and Sturve 2018). Besides, NP agglomerates/aggregates of heterogeneous morphology and size were identified in TEM images of the TiO_2_ NP stock dispersion. Sizes ranged from very small agglomerates/aggregates consisting of two to three NPs to 0.5 μm large agglomerates/aggregates (Fig. 1a) (Lammel and Sturve 2018). The presence of TiO_2_ NP agglomerates/aggregates was also confirmed by spICP-MS measurements performed on the stock (Fig. 1b). The most frequent size in the stock dispersion was 45.0 nm. The mean agglomerate size was 57.9 nm. The particle size-number frequency distribution is shown in Fig. 1b. The monomodal particle size-intensity distribution of TiO_2_ NPs was maintained after dilution in BSA solution and L-15 cell culture medium. The hydrodynamic size-frequency distribution by number of BSA-stabilized TiO_2_ NPs in L-15 medium at the beginning (*t* = 0) and the end of the exposure period (*t* = 24 h) determined by DLS is shown in Fig. 1c (left and right image, respectively). As apparent from this figure, a significant fraction of the NP agglomerates/aggregates (~ 50%) in L-15 medium maintained hydrodynamic diameters < 100 nm: The smallest agglomerates had a hydrodynamic diameter between 60 and 70 nm; the mean hydrodynamic diameter was ~ 100 nm. The hydrodynamic size distribution did not change in dependence of exposure time (24 h) and concentration (1–100 μg/ml) (Fig. 1c) (Lammel and Sturve 2018).

### Interaction of TiO_2_ NPs with the plasma membrane of RTL-W1 cells and endocytic uptake

**Fig. 2:**
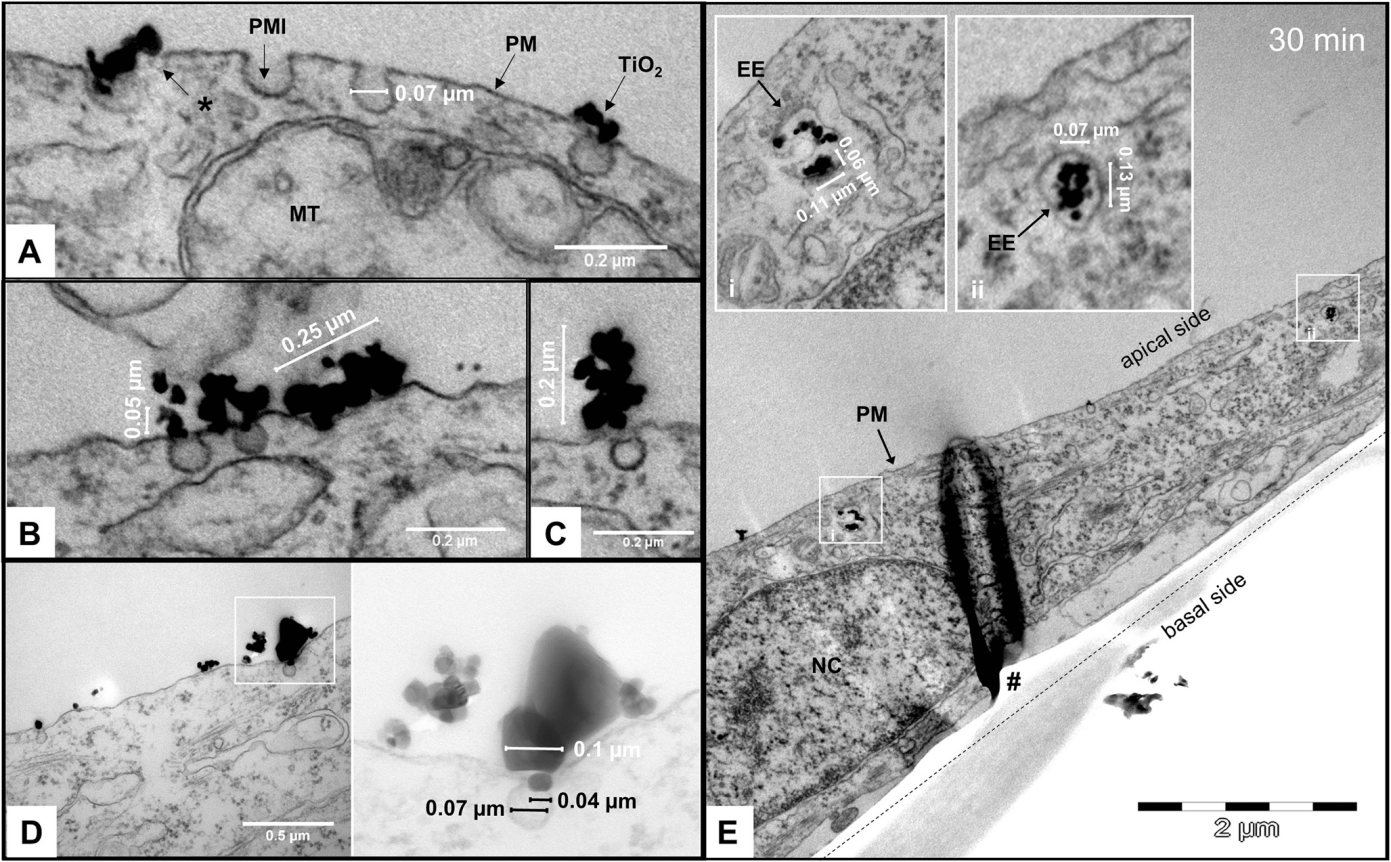
Interaction with the plasma membrane and endocytic uptake of TiO_2_ NPs. **a** Interaction of TiO_2_ agglomerates/aggregates with the outer leaflet of the plasma membrane accompanied by the formation of membrane invaginations. The asterisk (*) adverts to a comparatively smaller plasma membrane invagination, which was observed at the site of NP-membrane interaction. **b** and **c** TiO_2_ NP agglomerates/aggregates of comparable size (0.25 and 0.2 μm in **b** and **c**, respectively), but in different relative orientation to the plasma membrane (horizontal and vertical to the plane of the plasma membrane, respectively). **d** Induction of plasma membrane invagination by a large (> 100 μm) agglomerate/aggregate. The boxed-in area in **d** is displayed at higher magnification and different brightness and contrast at its right side. It demonstrates that the plasma membrane invagination occurs at the site where an individual NP being associated with the agglomerate/aggregates enters in contact with the cell surface. **e** TEM image taken 30 min after cell treatment showing TiO_2_ NP agglomerates/aggregates inside intracellular vesicles located in the apical cell periphery. The inserts (i and ii) in the upper left part of the image show the boxed-in areas at higher magnification. Sectioning was carried out perpendicular to the plane of the growth surface, which is indicated by the dotted line. The black structure in the middle of the micrograph, which is marked with a hashtag (#) is an artifact owed to a wrinkle in the ultrathin section. *PM* plasma membrane, *PMI* plasma membrane invaginations, *MT* mitochondrion, *EE* early endosome, *NC* nucleus. Scale bars: **a**, **b**, and **c** = 0.2 μm; **d** = 0.5 μm; and **e** = 2 μm

TEM analysis demonstrated individual TiO_2_ NPs and NP agglomerates/aggregates of different size, morphology, and relative orientation physically interacting with the outer leaflet of the plasma membrane (Fig. 2a–d). Concomitantly, plasma membrane invaginations were observed at the site of interaction on several occasions. The diameter of the endocytic vesicles budding inward from the plasma membrane was 0.07 ± 0.01 μm (mean ± SD, number of vesicles measured: *n* = 29). The majority of the NP agglomerates/aggregates, which were observed to induce vesicle formation, had a width (=minimum caliper diameter, or minimum Feret’s diameter) corresponding to or smaller than the vesicle’s diameter. Larger NP agglomerates/aggregates exceeding the vesicle’s dimension were also observed to induce vesicle formation when the interaction with the cell surface occurred via a smaller-sized appendage of the agglomerate/aggregate (Fig. 2c, d). Images of control cells are shown in Fig. S1 in Supplementary Information (SI).

TiO_2_ NP agglomerates/aggregates were identified inside intracellular vesicles. Evidence for internalization was observed at the first analysis time point, that is, 15 min after cell treatment. At this time point as well as at the second analysis time point, that is, 30 min after cell treatment, the NP-containing vesicles were predominantly located in proximity to the apical plasma membrane (Fig. 2e). The vesicles, or early endosomal compartments, contained one or more NP agglomerates/aggregates with dimensions of approximately 60–70 nm × 110–130 nm (Fig. 2e).

### Intracellular distribution and interaction with cellular organelles

**Fig. 3:**
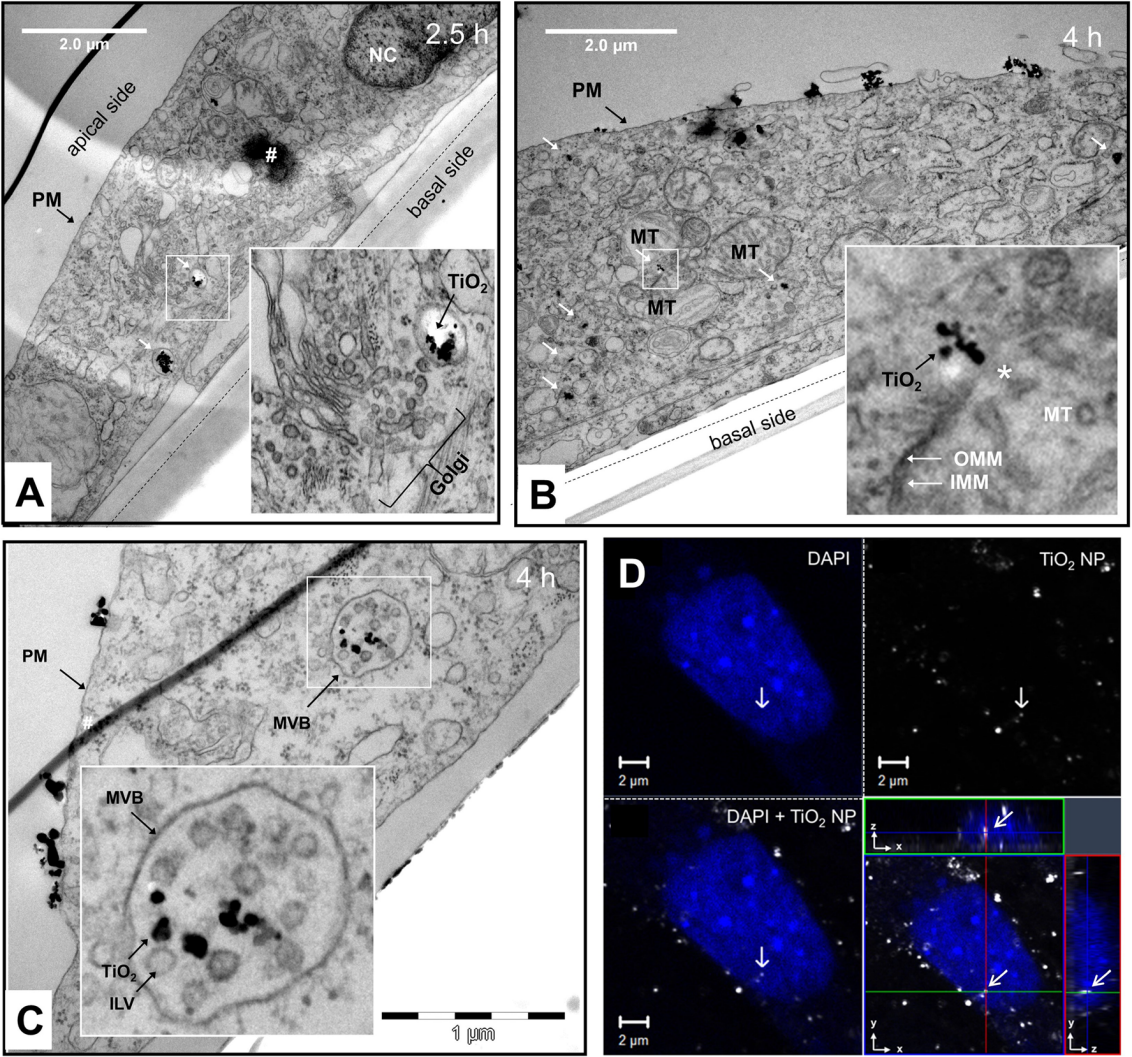
Intracellular fate and interaction with cellular organelles. **a** TEM image taken after 2.5 h showing TiO_2_ NP-containing vesicles in the basal cell periphery (white arrows). The boxed-in area is displayed at higher magnification in the lower right corner. It shows one of the NP-containing vesicles in close vicinity to the Golgi apparatus. **b** TEM image taken after 4 h showing TiO_2_ NP-containing vesicles distributed throughout the cytosol (white arrows). The boxed-in area is displayed at higher magnification in the lower right corner. It shows a TiO_2_ NP agglomerate/aggregate in close proximity (seemingly interacting) with the mitochondrial membrane(s). **c** TEM image taken 4 h after cell treatment showing TiO_2_ NPs inside an MVB. The boxed-in area is displayed as a close-up in the inset. **d** CLSM image of the nucleus of an RTL-W1 cell exposed to 100 μg/ml for 2 h. Upper left image: Nucleus stained with DAPI (shown in blue). Upper right image: TiO_2_ imaged in reflection mode (shown in white). Lower left image: Overlay of the upper left and right images. Lower right image: Focal plane (xy), in which the TiO_2_ NP was detected, together with xz- and yz-orthogonal sections along the green and red line, respectively. *PM* plasma membrane, *MVB* multivesicular body, *ILV* intraluminal vesicle, *NC* nucleus, *MT* mitochondrion, *OMM* outer mitochondrial membrane, *IMM* inner mitochondrial membrane. *Hashtag* indicates artifact in the section, *Asterisk* indicates part of MT in proximity to TiO_2_ NP agglomerate where IMM and OMM are not discernible. Scale bars: **a** and **b** = 2 μm, **c** = 1 μm, and **d** = 2 μm

With increasing incubation time (1, 2.5, and 4 h), the endocytosed NP were routed from the site of uptake, that is, from the apical plasma membrane to other regions in the interior as well as to the basal periphery of the cell (Fig. 3a). The TEM images showed NP-containing vesicles close to cellular organelles including the Golgi apparatus and mitochondria (Fig. 3a, b, respectively). On one occasion, a TiO_2_ NP agglomerate/aggregate was observed to interact with the mitochondrial membrane(s) (see inset in Fig. 3b). Furthermore, TiO_2_ NPs could be identified inside multivesicular bodies (MVBs) (Fig. 3c). Moreover, CLSM images showed the localization of TiO_2_ NPs in the nuclear periphery and seemingly inside the nucleus (Fig. 3d).

### Intracellular fate and accumulation

**Fig. 4:**
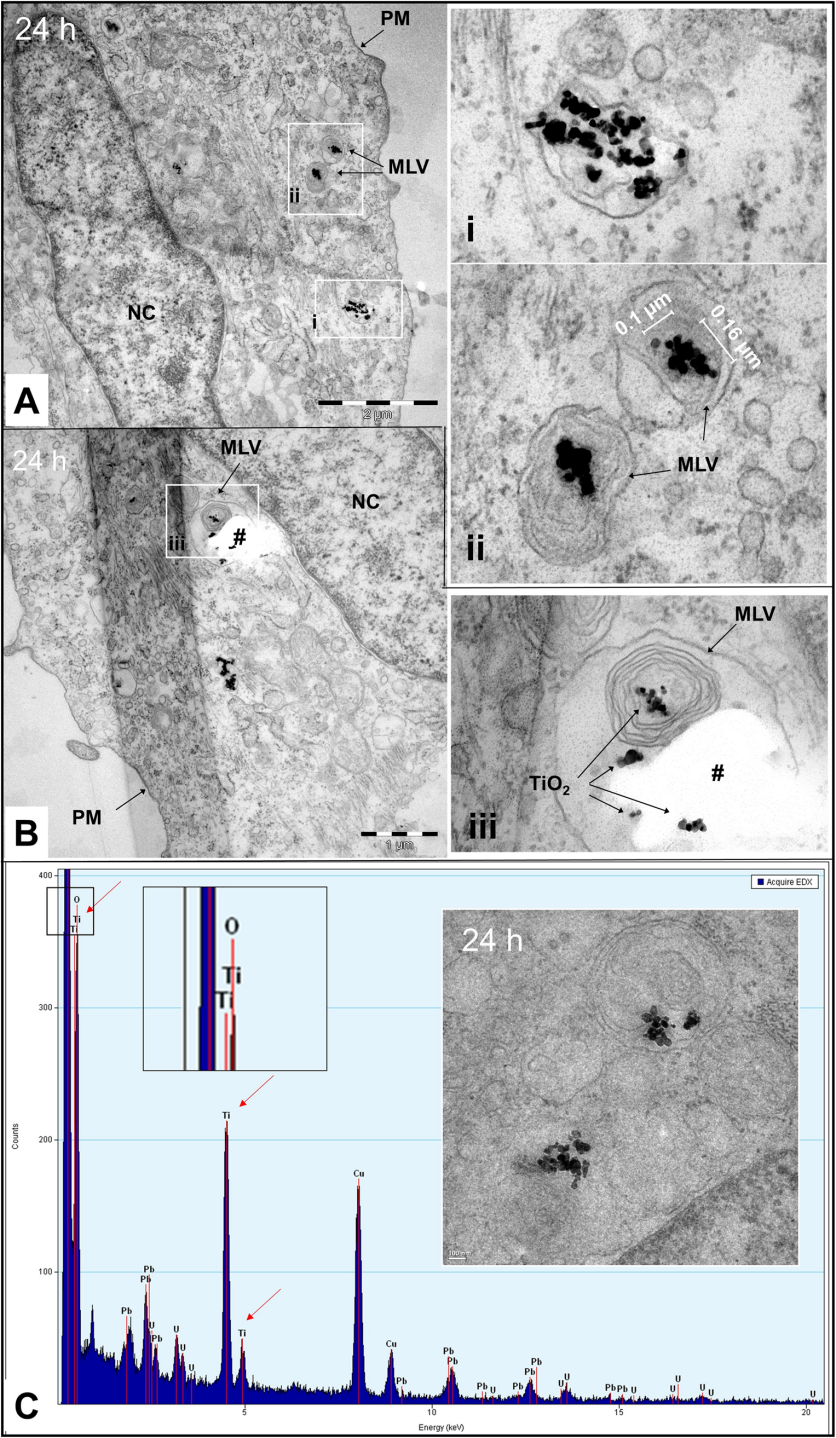
TiO_2_ NPs enclosed in multilamellar vesicles. **a** and **b** TEM images taken from two different cells, which have been exposed for 24 h showing TiO_2_ NP agglomerates/aggregates inside of vesicles with more than one delimiting membrane (multilamellar vesicles, MLVs). The inserts i, ii, and iii display the boxed-in areas in **a** and **b** at higher magnification. Insert i shows a vesicle with two limiting membranes containing various loosely packed NP agglomerates/aggregates. Insert ii shows two MLVs with three to four limiting membranes containing one compact NP agglomerates/aggregate each. Insert iii shows a large MLV with six (or more) concentric membranes forming different sub-compartments. The white spot in insert iii, which is marked with a hashtag (#), is a hole in the ultrathin section presumably caused by ripping out a large agglomerate/aggregate during sectioning. **c** EDX spectrum confirming that the electron-dense objects seen in TEM images correspond to TiO_2_. Ti peaks were indicated with arrows. The boxed-in area (black frame) is shown enlarged to facilitate identification of the elements that were assigned to the peaks (Ti and O). The area to which the EDX spectrum corresponds is shown in the inset. *PM* plasma membrane, *NC* nucleus, *MLV* multilamellar vesicle. Scale bars: **a** = 2 μm, **b** = 1 μm, and **c** (TEM image) = 100 nm

Uptake of TiO_2_ NPs into endocytic vesicles continued over the length of the experiment (24 h) resulting in TiO_2_NP accumulation inside the cell. The size of the endosomal compartments and the amount of TiO_2_ per compartment increased with time. Furthermore, TiO_2_ NP-containing vesicles with more than one delimiting membrane, in this article referred to as multilamellar vesicles (MLVs), were observed. This kind of vesicle was first observed after 2.5 h (Fig. S2) and was more frequent after 24 h (Fig. 4). The morphology of the MLVs, such as their size and number of delimiting membranes, and the NP agglomerates/aggregates enclosed therein, such as their size, compactness, and number, were fairly heterogeneous (compare inserts i, ii, and iii in Fig. 4a, b). Elemental analysis of TEM sections using EDX analysis demonstrated a high relative abundance of Ti (compared to regions without NPs) providing evidence that the identified electron-dense objects correspond to the tested nanomaterial (i.e., TiO_2_ NPs) (Fig. 4c). It must also be remarked that all other factors including the size- and shape-resemblance with TiO_2_ NPs in the stock dispersion, the localization of the particles *inside* the resin section (i.e., *not on to top* of the section, which would be the case if the electron-dense material corresponded to precipitates of applied post-stains), as well as the localization *inside* vesicles (i.e., *not* randomly scattered on top of the section) excludes the possibility that the imaged particles correspond to anything else but the applied nanomaterial.

### Quantitative analysis of TiO_2_ NP uptake in RTL-W1 using mass spectrometry

**Fig. 5:**
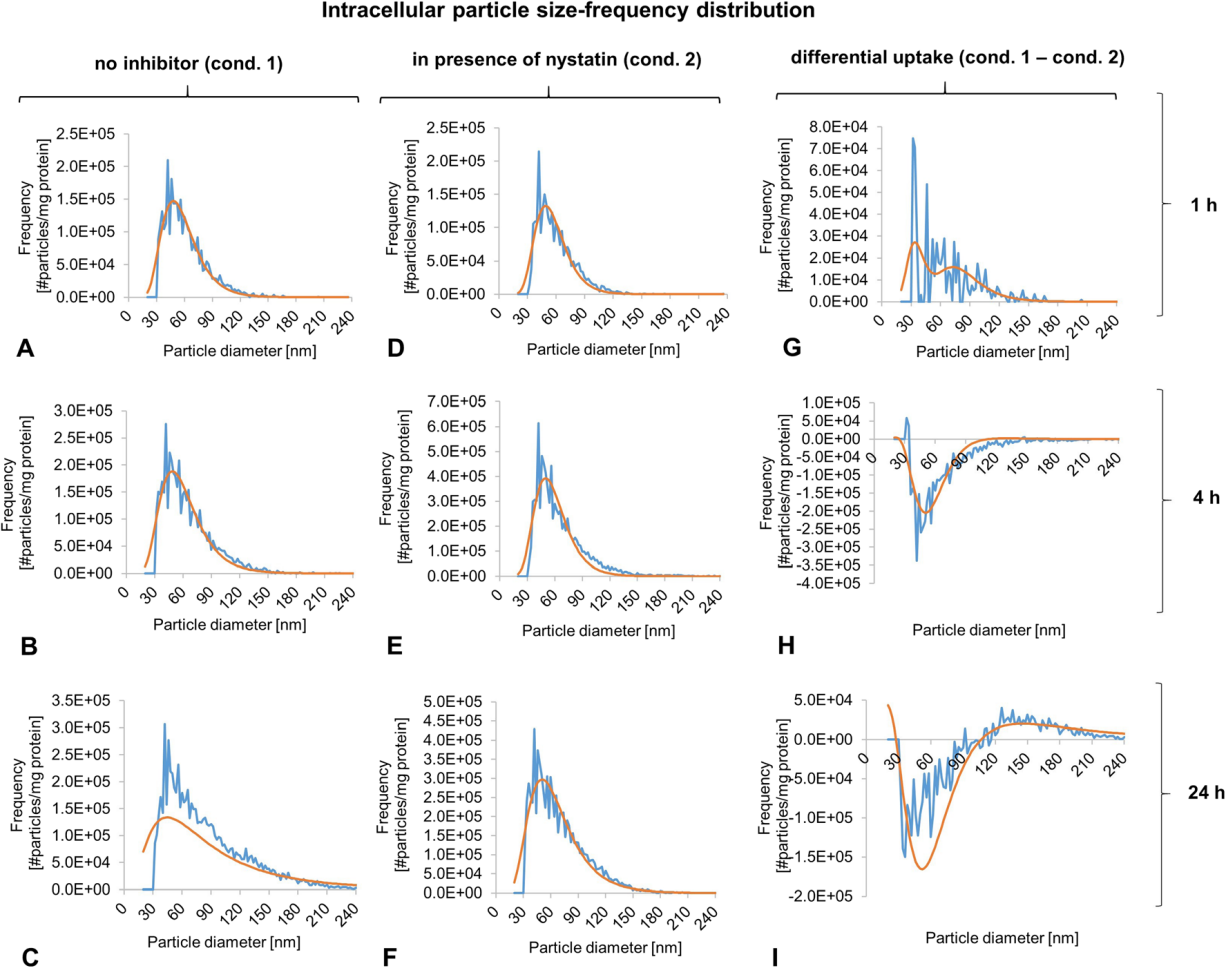
Intracellular particle size-frequency distribution of TiO_2_ NPs. Particle size-frequency distribution of TiO_2_ NPs inside RTL-W1 cells determined by spICP-MS following 1, 4, and 24 h of exposure (top, middle, and bottom rows, respectively) to 10 μg/ml TiO_2_ NPs in the absence (**a**–c) and presence (**d**–**f**) of 50 μM nystatin. Smooth curves (displayed in orange color) show the lognormal fits. The LOD (size) was 32 nm. **g**–**i** show the relative difference in the intracellular TiO_2_ particle size-frequency distributions between both treatments (=particle size-frequency distribution without inhibitor − particle size-frequency distribution with inhibitor)

Table 2 and Fig. 5 show the intracellular Ti content, particle number, and particle size distribution quantified by spICP-MS following 1, 4, and 24 h of exposure to TiO_2_ NP in the absence and presence of nystatin. Note that the use of a bulk TiO_2_ as a reference material was not required in this study as results demonstrated that endocytic uptake was limited to particle sizes in the nanometric range (vesicle diameter ~ 70 nm). TiO_2_ NP agglomerates > 100 nm present in the dispersion used for cell exposures were not taken up by RTL-W1 cells. A size > 100 nm corresponds to the size of bulk material, which often is in the sub-micron to micron range.

**Table 2:** Summary of spICP-MS results

Treatment	Most freq. size (nm)	Mean size (nm)	Dissolved conc (μg Ti/L)	Total particle conc (#parts/mL)	Total mas conc (μg Ti/L)	Protein content normalized particle conc (#parts/mg)	Protein content normalized mass conc. (ngTi/mg)
1 h TiO_2_	48	60	<LOD	8.88E+07	35.24	1.03 + 08	4.09E+01
4 h TiO_2_	48	64	<LOD	1.18E+08	61.24	1.28 + 08	7.10E+01
24 h TiO_2_	55	84	<LOD	2.31E+08	333.35	3.13 + 08	3.87E+02
cntrl (24 h)	<LOD	<LOD	<LOD	<LOD	<LOD	ND	ND
1 h TiO_2+_ nystain	48	60	<LOD	8.59E+07	30.75	1.22 + 08	3.56E+01
4 h TiO_2+_ nystain	50	65	<LOD	1.79E+08	102.85	1.58 + 08	1.19E+02
24 h TiO_2+_ nystain	50	68	<LOD	2.35E+08	149.74	2.81 + 08	1.74E+02
cntrl (24 h) + nystain	<LOD	<LOD	<LOD	<LOD	<LOD	ND	ND

spICP-MS: LOD (size) = 32 nm, LOD (dissolved conc.) = 0.1 μg/L

*ND* not determined

The results obtained by spICP-MS confirmed our observations made by TEM. They provided quantitative evidence that TiO_2_ NPs were taken up and accumulated inside RTL-W1 cells with time (Table 2). Linear regression analysis gave an approximate uptake rate of 15.3 ng Ti/mg total cellular protein content/h (*r*^2^ = 0.998) or 9 × 10^6^ TiO_2_ NP agglomerates/mg total protein content/h (*r*^2^ = 0.999) (Fig. S3). The intracellular dose after 24 h exposure was ~ 3.9 × 10^2^ ng Ti/mg protein and ~ 3.1 × 10^8^ particles/mg protein, respectively (Table 2). The total mass measured inside the cells collected after 24 h exposure was 333.35 ng Ti corresponding to approximately 1% of the total Ti mass applied to the cell culture dish (~ 36 μg Ti; cp. “Material and Methods” section).

Furthermore, spICP-MS demonstrated that NPs/NP agglomerates further increased in size *after* being taken up (Table 2, Fig. 5a–c). The most frequent intracellular agglomerate/aggregate size was 48, 48, and 55 nm after 1, 4, and 24 h of exposure, respectively. The mean intracellular agglomerate/aggregate size increased from 60 nm (1 h) to 84 nm (24 h) (Table 2). This shift in the intracellular particle size-frequency distribution towards larger diameters can be particularly well discerned comparing Fig. 5a, c (note that after 1 h nearly all intracellular agglomerates still have a size ≤ 150 nm, while after 24 h there is a considerable fraction of agglomerates with a size in the range of 150–250 nm).

In addition, it was observed that cells exposed to TiO_2_ NPs in the presence of the caveolae-mediated endocytosis inhibitor nystatin took up a lower number of NP agglomerates in the size range of ~ 30–40 nm and ~ 60–90 nm large, compared to cells not co-exposed to nystatin (shown by the two frequency peaks in Fig. 5g; note that the LOD (size) was 32 nm). The size range excluded from the nystatin treatment corresponded to the size range of small NP agglomerates, which were previously observed to be taken up into vesicles budding inwards from the plasma membrane (see TEM images in Fig. 2). This shows that nystatin inhibits TiO_2_ NP internalization via these vesicles (caveolae). Moreover, cells, where caveolae-mediated endocytosis was inhibited, had a considerably lower intracellular Ti content (~ 45% after 24 h; mass concentration) showing that TiO_2_ NP uptake occurred at a lower rate compared to cells, where this uptake pathway was not inhibited (Table 2). The number of TiO_2_ NPs present inside cells treated with inhibitor was lower as well, but the differences compared to the inhibitor-free exposure were not as strongly pronounced as for the data expressed as mass concentrations (Table 2). That mass concentration and particle number concentration were not congruent can be explained by our previous observation that several endocytosed NP agglomerates are concentrated in common intracellular compartments where they then interact with each other forming larger agglomerates (Fig. 4). This did not seem to happen in nystatin-treated cells: Fig. 5d–f show that no shift in the intracellular particle size-frequency distribution towards larger diameters occurred between early (1 h) and later (24 h) analysis time points.

It must be remarked that at the first two measurement time points, that is, after 1 and 4 h of exposure, the differences in mass concentrations and particle number concentrations between control cells (i.e., cells exposed to NPs, but not to nystatin) and nystatin-exposed cells were not as prominent as after 24 h. In fact, in cells exposed for 4 h, the intracellular Ti concentration was even slightly higher in the presence of the inhibitor.

### Effects on cellular redox homeostasis

**Fig. 6:**
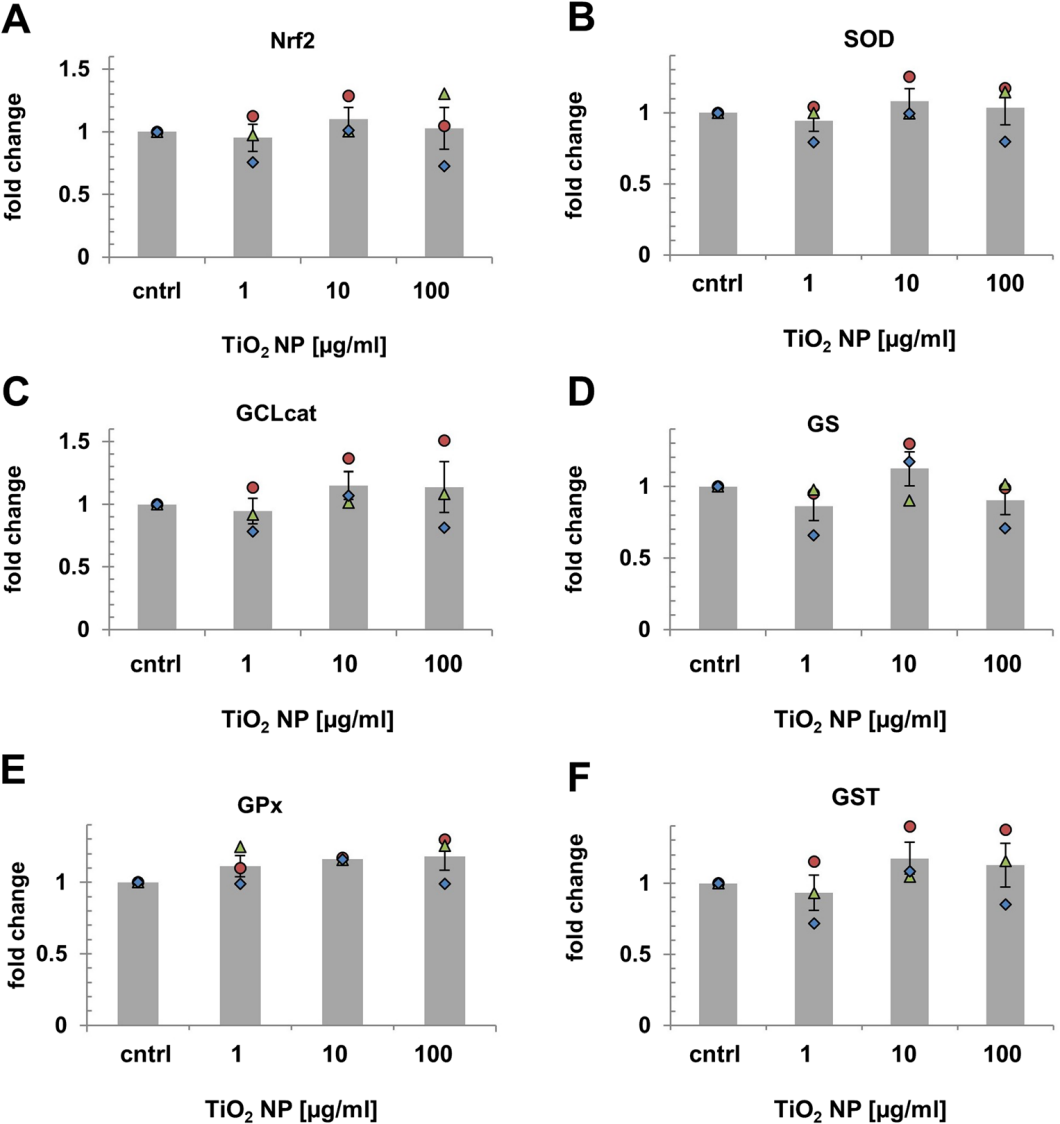
Effects on Nrf2/ARE signaling. Relative mRNA expression levels of Nrf2 (**a**), SOD (**b**), GCLcat (**c**), GS (**d**), GPx (**e**), and GST (**f**) displayed as fold change (FC) of control. Markers in each graph (circle, triangle, diamond) show the FC (=2^-ddCq) observed in the first, second, and third independent experimental repetitions (*n* = 3). Bars and error bars correspond to the FC means and their standard errors. No statistically significant differences (one-way RM ANOVA) were observed

No statistically significant differences in expression levels of oxidative stress-related genes were observed following RTL-W1 cells exposure to 1, 10, and 100 μg TiO_2_ NP/ml for 24 h compared to the medium control (Fig. 6). However, a slight trend towards a dose-dependent upregulation was observed in at least one of the three experimental repetitions for SOD-1, GCLcat, GPx, and GST, with mRNA expression levels being increased between 1.1- and 1.5-fold at 100 μg/ml TiO_2_ NP (red circles, Fig. 6).

## Discussion

### Interaction of TiO_2_ NPs with the plasma membrane

In the present study, we observed that the sites where TiO_2_ NPs interacted with the cell surface often featured invaginations of the plasma membrane. Figure 2a, for instance, shows a NP agglomerate/aggregate that interacted with the plasma membrane at two positions. At both sites of interaction, plasma membrane invaginations were observed, one of which (the one marked with an asterisk) was still relatively small, suggesting that it was still in the process of formation. This suggests that plasma membrane invaginations were *induced* by the NPs.

Interestingly, not all TiO_2_ NPs interacting with the cell surface elicited this response. Several TEM images suggest that the formation of plasma membrane invaginations depended on the agglomerate’s shape and relative orientation to the cell surface. For instance, Fig. 2b, c show that of two NP agglomerates of comparable size (~ 0.2–0.25 μm), of which one is horizontally and the other perpendicularly oriented to the cell surface, only the latter induces plasma membrane invagination. This suggests that initiation of vesicle formation depends on the particle’s *apparent* size (i.e., how the particle’s size is perceived by the cell). Yet, a NP/agglomerate’s ability to induce plasma membrane invagination does not necessarily lead to its internalization, as NPs/agglomerates with at least two dimensions larger than the invagination’s diameter (here 0.07 ± 0.01 μm) cannot be taken up into the forming vesicle. These observations are consistent with previous studies showing that NP uptake into endocytic vesicles may depend on the particles’ surface orientation and size (Dasgupta et al. 2014, Herd et al. 2013). The question arises whether the mere induction of vesicle formation could already translate into alterations of cellular processes, as the former is concomitant with changes in the localization, organization, and activity of a variety of structural, regulatory, and signaling proteins (Bryan et al. 2013, Chidlow and Sessa 2010).

### Endocytic uptake mechanism of TiO_2_ NPs in RTL-W1 cells

TiO_2_ NPs were taken up into endocytic vesicles, which lacked the distinct coat that is characteristic for clathrin-coated vesicles (Pavelka and Roth 2005; Reifarth et al. 2018), excluding clathrin-dependent endocytosis as uptake pathway. The scientific literature knows several clathrin-*in*dependent mechanisms including RhoA-, CDC42-, ARF6-, and flotillin-mediated endocytosis (Mayor and Pagano 2007), but our results strongly suggest that TiO_2_ NP uptake by RTL-W1 cells occurs via caveolae-mediated endocytosis. First, the NP-induced plasma membrane invaginations match the typical ultrastructural appearance of caveolae, that is, smooth flask-shaped invaginations of the plasma membrane with diameters ranging from 50 to 100 nm. Besides, caveolae-mediated endocytosis can be induced by the cargo itself (Hillaireau and Couvreur 2009), which is consistent with our observation described above. Furthermore, caveolae-mediated endocytosis is known to be involved in the uptake and transcellular transport of various biomolecules including albumin (Schnitzer 2001), with which TiO_2_ NPs were coated in our study. Albumin is recognized by a membrane receptor termed gp60 concentrated in caveolae, and its binding triggers the phosphorylation of caveolae-associated proteins caveolin-1 and dynamin-2 resulting in the internalization and fission of caveolae from the plasma membrane (Minshall et al. 2003). This process is best studied in endothelial cells, but also occurs in other cell types. For instance, Botos et al. (2008) demonstrated that albumin is able to induce the formation and internalization of caveolae in human hepatoma cells, and that immunogold-labeled albumin is taken up via this pathway. Little is known about caveolae-mediated endocytosis and albumin-uptake in piscine liver cells, but it is well possible that RTL-W1 cells express caveolae-associated membrane receptors ortholog to gp60, which are able to bind albumin-coated TiO_2_ NPs mediating uptake into caveolar membrane invaginations. That protein corona-membrane receptor interactions may play an important role in TiO_2_ NP (E171) internalization was also suggested by Krüger et al. (2017). Further evidence for caveolae-mediated uptake of TiO_2_ NP in RTL-W1 cells is provided by our spICP-MS data, which showed considerably lower Ti accumulation in the presence of the caveolae-mediated endocytosis inhibitor nystatin. Besides, the size of particle population that was excluded from uptake in the presence of the inhibitor matched the size of the particle sub-population observed to be taken up into the endocytic vesicles (Table 2, Fig. 5g). Unfortunately, no literature on TiO_2_ NP Aeroxide® P25 uptake in piscine cells is available for comparison, but our results are consistent with findings in mammalian cells (Caco-2 cells) showing that caveolae-mediated endocytosis is involved in the uptake of TiO_2_ NP Aeroxide® P25 (Gitrowski et al. 2014). The pathway has also been suggested to be involved in the uptake of other TiO_2_ NPs (synthesized, different primary sizes), for instance, in astrocyte-like cells (Hsiao et al. 2016b) and prostate cancer PC-3M cells (Thurn et al. 2011).

### Uptake kinetics, intracellular fate, and accumulation of TiO_2_ NPs

After 15–30 min, TiO_2_ NPs were identified inside early endosomes (EEs) located in proximity to the apical plasma membrane (Fig. 2e). The presence of more than one NP agglomerate inside EEs suggests that the latter received material from several endocytic vesicles (Fig. 2e, insert i). This observation is in agreement with previous studies showing that caveolae-mediated intracellular transport of cargo is a rapid process (Oh et al. 2007). It is difficult to identify the exact further intracellular route of endocytosed TiO_2_ NPs solely based on information obtained by TEM, but our images suggest that they are trafficked through different intracellular compartments.

For instance, TiO_2_ NPs were found inside multivesicular bodies (MVBs), which are vesicular vacuoles that detach or mature from EEs and are characterized by several intraluminal vesicles (ILVs) containing proteins destined for degradation (Katzmann et al. 2002). After their formation, MVBs acidify and fuse with late endosomes (LEs) delivering them their cargo (Scott et al. 2014). LEs in turn fuse with lysosomes (LYs) forming a transient hybrid organelle, which then matures into classical secondary LYs. Thus, the presence of TiO_2_ NPs inside MVBs strongly suggests that they are routed to the cell’s degradative compartments. That TiO_2_ NPs may end up in lysosomes was previously observed in mammalian cell models (Kruger et al. 2017; Zhu et al. 2012). However, one needs to bear in mind that MVBs (and late endosomes) do not *always* deliver their cargo to LYs. In many cell types, they can fuse again with the plasma membrane and release their content including ILVs into the extracellular space (Raposo and Stoorvogel 2013; Scott et al. 2014). ILVs released via this way are referred to as exosomes and play an important role in intercellular communication by transferring proteins, lipids, and RNA to nearby and remote cells (Harding et al. 2013; Raposo and Stoorvogel 2013), raising the question whether NPs enclosed in ILVs could share the same fate. From our images, it is difficult to discern whether the TiO_2_ NPs were located between or inside the ILVs. ILVs originate from luminal invaginations of endosomal membrane domains, that is, their interior is topologically equivalent to the cytosol. Thus, the presence of NP inside ILVs could only be explained if some of them had directly translocated over the plasma membrane or escaped from intracellular compartments (Nakamura and Watano 2018). However, except for the agglomerate in close vicinity, seemingly interacting with the mitochondrial membrane(s) (Fig. 3b), we found no evidence for cytosolic location. Further experiments specifically examining NP uptake into ILVs/release with exosomes were out of the scope of this study, but are planned for the future.

Also, our TEM images showed that the amount of TiO_2_ NPs inside intracellular vesicular compartments increased with time, suggesting that agglomerates endocytosed at different times or sites of the plasma membrane were routed to one or more common endo-lysosomal compartments. Furthermore, NP confinement and accumulation in intracellular vesicles appeared to be concomitant with a further increase in agglomerate size (Fig. 5a–c). These observations are consistent with findings by Chernenko et al. (2009), Pan et al. (2009), and Tomankova et al. (2015) that NPs can further agglomerate/aggregate intracellularly.

TEM analysis revealed that many TiO_2_ NP agglomerates ended up in multilamellar vesicles (MLVs). Localization of TiO_2_ NPs inside lamellar bodies was also reported by Andersson et al. 2011. MLVs were described in various cell types including fish hepatocytes and can have different origin and function, and occur under normal, stressed, or pathological conditions (Aleš Iglič and Kralj-Iglič 2015; Anken et al. 2004; Arnold et al. 1996; Asztalos et al. 1990; Burkhardt-Holm et al. 1999; Cox et al. 1988; Drobne et al. 2008; Hugla and Thomé 1999; Marchetti et al. 2004; Samie and Xu 2014). For instance, they can develop from LEs, which accumulate internal membrane material adapting pleomorphic and complex ultrastructures (including onion-like sheet organization) along the degradation pathway to lysosomes (Bissig and Gruenberg 2013; Chevallier and Gruenberg 2006; Vacca and Gruenberg 2016; Huotari and Helenius 2011). Furthermore, MLVs can form when extra- or intracellular material is autophagocytosed (Vacca and Gruenberg 2016; Hariri et al. 2000; Lucocq and Walker 1997). Although the exact origin of the MLVs observed in our study remains to be elucidated, it is likely that their formation reflects an attempt of the cell to confine the intracellularly accumulating NPs for later degradation. However, considering its high chemical inertness, the question arises whether RTL-W1 cells (and fish liver cells in general) will be able to degrade and eliminate TiO_2_ NPs or if accumulation proceeds until vesicle and cellular overload occur. This question is particularly interesting against the backdrop of results showing that TiO_2_ NPs having accumulated in rainbow trout liver were not entirely eliminated during depuration (Ramsden et al. 2009).

In our study, intracellular TiO_2_ NP accumulation seemed to follow linear kinetics (Fig. S3). This is expected when uptake occurs actively and no counteracting processes, such as export or “dilution” by cell division, set in—assuming continuous exposure to excess concentrations and stable particle size distribution in the exposure medium (Aberg et al. 2013; Kim et al. 2012; Laomettachit et al. 2017). Based on our results (see section the “Quantitative analysis of TiO2 NP uptake in RTL-W1 using mass spectrometry” section), the intracellular load after 24 h exposure was approximately 0.013–0.13 pg Ti or ~ 10–100 particles (here: agglomerates) per cell when literature data on the approximate total protein content of single cells (~ 30–300 pg) is used for calculations (Cheung et al. 2013). These values are in good agreement with those measured by Hsiao et al. (2016a) in mouse neuroblastoma cells (0.36–1.93 pg/cell) after 24 h exposure to synthesized TiO_2_ NPs applied at the same concentration (10 μg/ml)—especially considering that mouse cells were cultured at 37 °C (i.e., have a higher metabolic rate), and other experimental parameters, such as NP agglomeration and settling behavior varied between their and our system.

### Interaction with cellular organelles

To the best of our knowledge, this study provides the first (visual) evidence for a possible interaction of TiO_2_ NPs with mitochondria (Fig. 3b). Interestingly, the NP agglomerate appeared not to be enclosed in an endocytic vesicle, but freely located in the cytosol as previously observed for other nanomaterials Lammel and Navas (2014). This observation may suggest that some of the endocytosed NPs were able to escape from the endosomal-lysosomal system. It remains to be elucidated whether such interaction occurs frequently or represents an exceptional event, but it gives rise to concern, as structural damage of mitochondria can lead to oxidative stress (Manke et al. 2013).

TiO_2_ NP-containing vesicles were also found in the perinuclear region in proximity to the nuclear envelope. CLSM images further suggested that some TiO_2_ NPs might be able to enter the nucleus (Fig. 4d), which was also suggested by Hackenberg et al. (2010), Shukla et al. (2011), and Chan et al. (2011). NPs can get into the interior of the nucleus by transport through nuclear pores or by accidental enclosure when the nuclear envelop de- and then reassembles again in the course of mitosis. The nuclear pore complex has an inner diameter of ~ 40 nm (Kabachinski and Schwartz 2015), that means individual (protein-coated) TiO_2_ NPs would hypothetically be able to pass through if they were located in the cytosol. Nuclear import of macromolecules is a complex process, which is very selective and highly controlled. Thus, passive diffusion and accidental active transport seems unlikely. Furthermore, the imaged particle is probably a larger agglomerate, as TiO_2_ particles ≤ 40 nm probably have a too low light-scattering power to be visualized in reflection mode. The second option, that is, enclosure during nucleus re-assemblage, also seems unlikely, considering that exposure was conducted using confluent cell cultures showing limited proliferative activity. Besides, nuclear localization was observed as early as 2 h after treatment, and RTL-W1 cells have a cell cycle time > 24 h. On the other hand, this may explain why TiO_2_ NPs were not identified in the nucleus more often. Bearing the above discussion in mind, one may also need to consider the possibility that the image is misleading and the TiO_2_ NPs were situated in a nuclear fold (outside the nucleus) like it was observed for TiO_2_ NP agglomerates in Pan et al. 2009.

### Effect of TiO_2_ NP accumulation on cellular redox homeostasis

Cell exposure to NPs including TiO_2_ was reported to result in reactive oxygen species (ROS) formation through NP surface catalyzed reactions and NP-induced dysfunction of cellular organelles (Auffan et al. 2011; Chan et al. 2011; Jaeger et al. 2012; Jiang et al. 2008; Manke et al. 2013; Park et al. 2008; Reeves et al. 2008; Setyawati et al. 2013; Stern et al. 2012; Zhu et al. 2012). The close proximity of TiO_2_ NPs to mitochondria and their accumulation in endo-lysosomal compartments prompted us to examine if exposure resulted in perturbation of cellular redox balance measuring mRNA expression levels of Nrf2-controlled genes which are often upregulated as the first line of defense to restore homeostasis. Our results suggest that TiO_2_ NP accumulation does not lead to perturbation of cellular redox homeostasis in RTL-W1 cells—at least during acute exposure. A possible reason for this could be the confinement of TiO_2_ NPs in MLVs or a protective effect of the NPs’ BSA-corona (Runa et al. 2016; Wang et al. 2013). The assessment of the long-term fate and effects of intracellular accumulated TiO_2_ NPs was out of the scope of this work but should be investigated in future studies.

## Conclusions

In this study, we describe in detail the interaction, uptake, and intracellular fate of one of the most frequently studied TiO_2_ NPs (Aeroxide® P25) in fish liver cells (RTL-W1 cells). Our results showed that the physical interaction of serum albumin-coated TiO_2_ NPs with the plasma membrane induces their uptake via a clathrin-independent and nystatin-sensitive mechanism—most probably caveolae-mediated endocytosis. TEM images and spICP-MS data on the intracellular particle size-frequency distribution demonstrated that the diameter of endocytic vesicles (~ 70 nm) forming at the plasma membrane level determines which NP size fraction is taken up by the cells. NP agglomerates > 100 nm present in the exposure medium are excluded from uptake. Thus, the relative NP size-frequency distribution in medium dispersions used for cell exposures is a factor that may critically influence intracellular NP accumulation and hence the effective cellular exposure dose (intracellular NP burden). Also, our results show that NP/agglomerate size may increase intracellularly, that is, *after* their uptake. The size increase is probably a consequence of the transport and confinement of the endocytosed NPs/agglomerates in common intracellular compartments (MLVs), where they are concentrated and further agglomerate. Furthermore, we did not observe any perturbation of cellular redox homeostasis or toxicity. Future studies need to determine if TiO_2_ NPs can be eliminated again and if a continuous intracellular accumulation of the inert material adversely affects the performance of cellular processes.

## Electronic supplementary material

Figure S1 TEM images of the control. RTL-W1 cells exposed to L-15 containing BSA but no TiO_2_ NPs. Scale bars: A = 500 nm, B and C = 1 μm, D = 2 μm (JPG 1616 kb)

Figure S2 MLVs containing TiO_2_ NP agglomerate in RTL-W1 cell exposed for 2.5 (PNG 28808 kb) (PNG 28808 kb)

High Resolution Image (TIF 4097 kb)

Figure S3 Time-dependent uptake of TiO_2_ NPs in RTL-W1 cells. A) Increase in intracellular Ti concentration. B) Increase in particle number. Dotted line: trend line of linear regression. The corresponding equations and coefficients of determination (r^2^) are displayed in the plot area. (JPG 297 kb)
