# Supplementary figures and images for: Endocytosis, intracellular fate, accumulation, and agglomeration of titanium dioxide (TiO2) nanoparticles in the rainbow trout liver cell line RTL-W1
Source: Environ Sci Pollut Res Int. 2019 Mar 31;26(15):15354–72. doi: 10.1007/s11356-019-04856-1 (PMC6529399; doi:10.1007/s11356-019-04856-1)

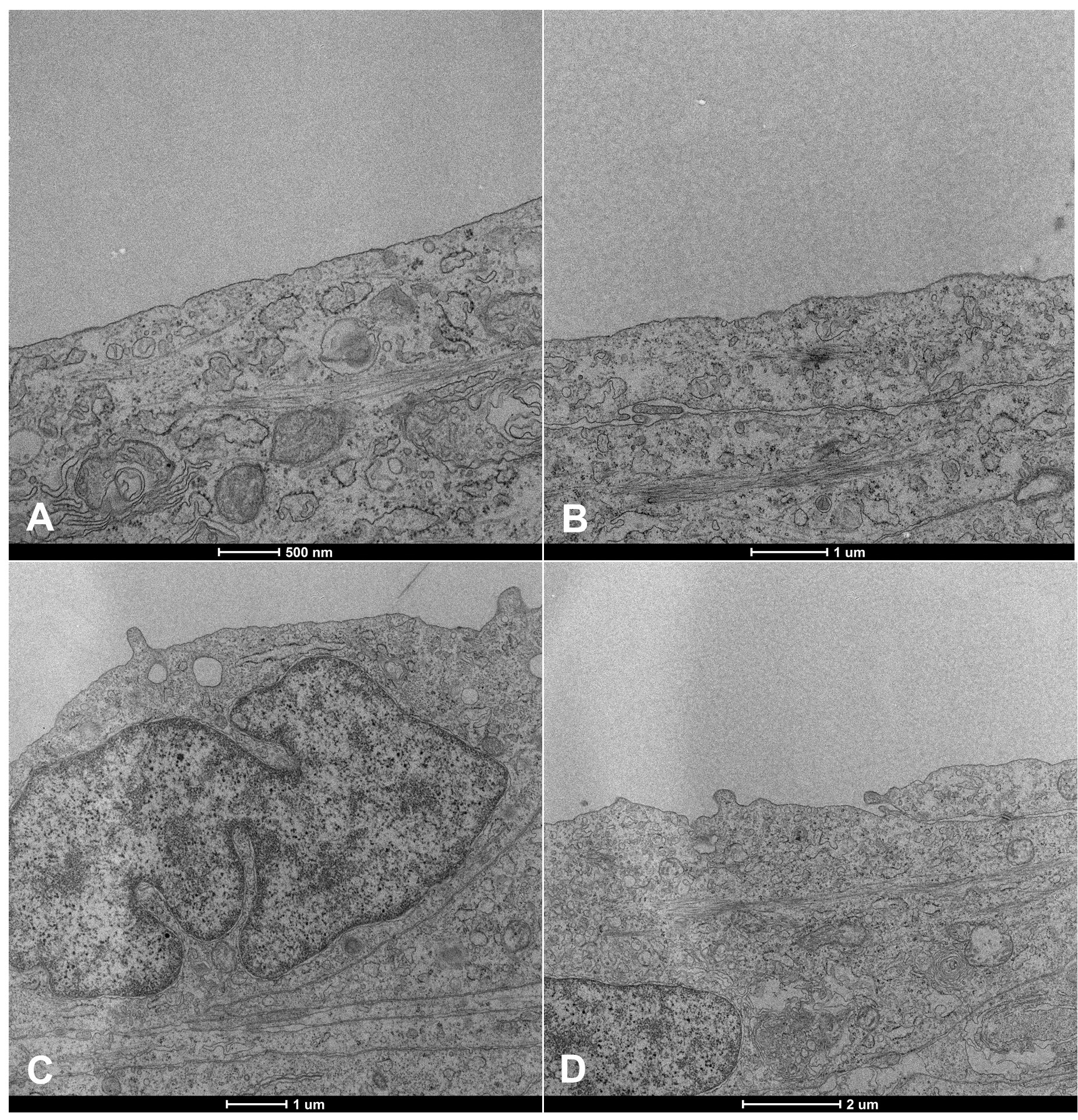

Supplement: Supplementary file 1 — TEM images of the control. RTL-W1 cells exposed to L-15 containing BSA but no TiO2 NPs. Scale bars: A = 500 nm, B and C = 1 μm, D = 2 μm (JPG 1616 kb) [file 11356_2019_4856_MOESM1_ESM.jpg]

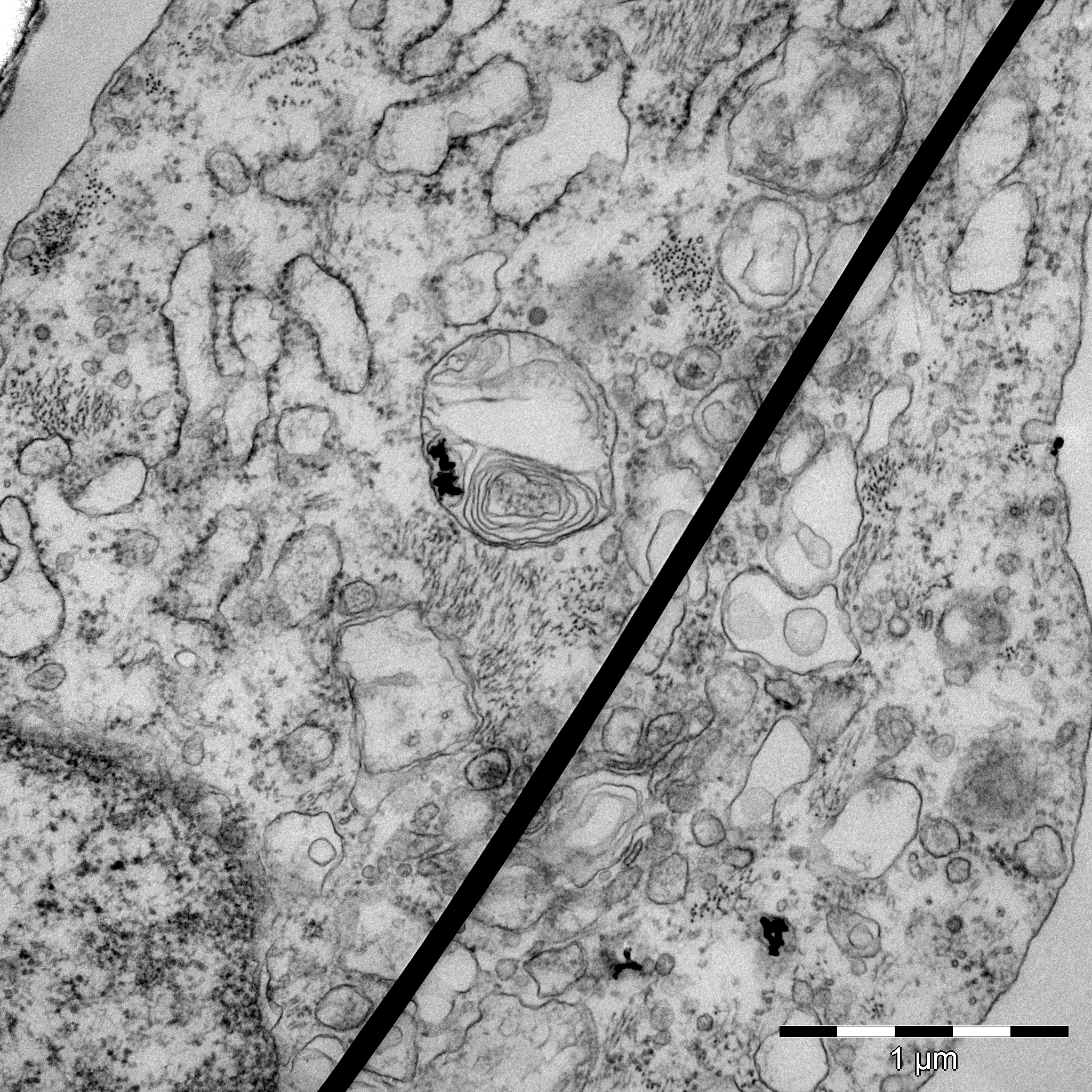

Supplement: Supplementary file 3 — High Resolution Image (TIF 4097 kb) [file 11356_2019_4856_MOESM2_ESM.tif]

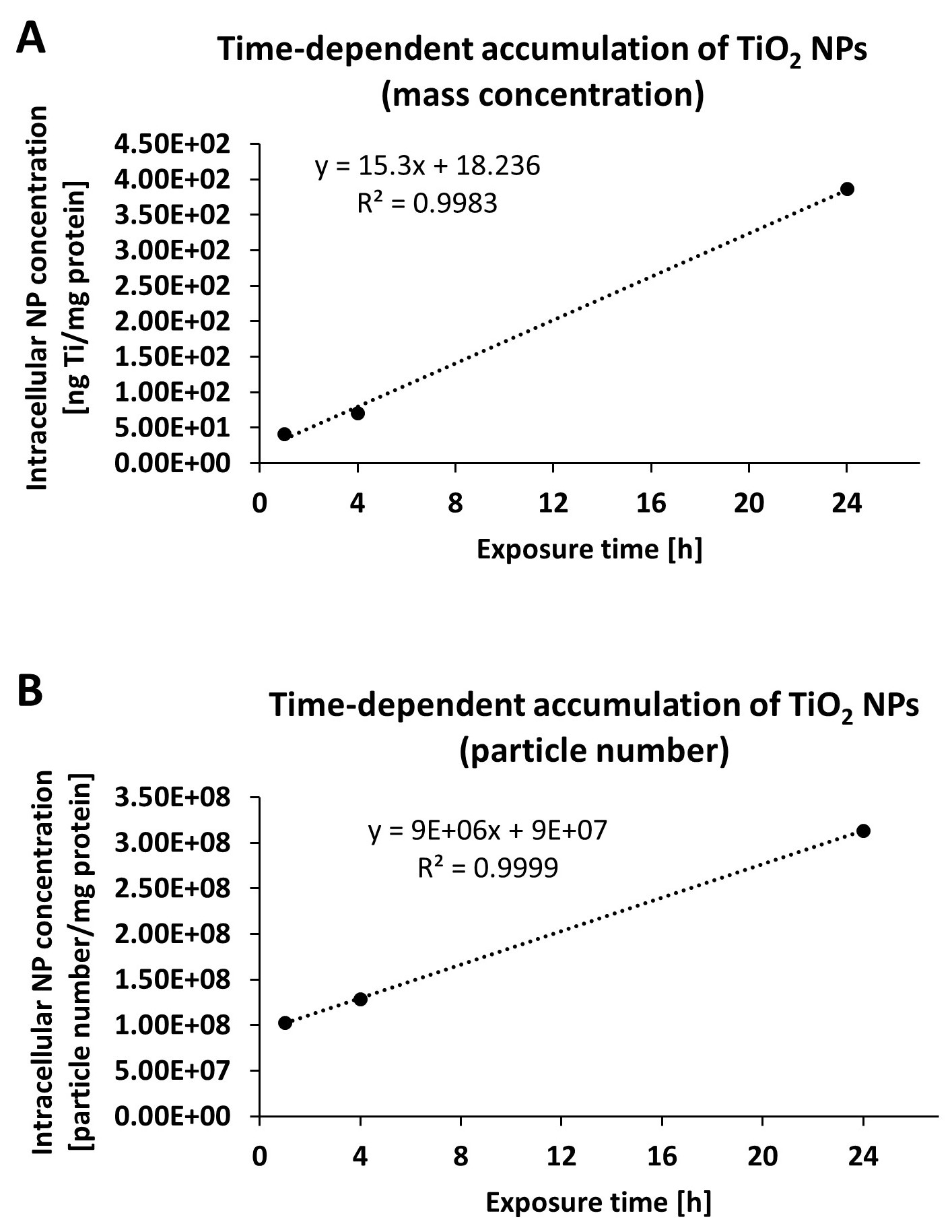

Supplement: Supplementary file 4 — Time-dependent uptake of TiO2 NPs in RTL-W1 cells. A) Increase in intracellular Ti concentration. B) Increase in particle number. Dotted line: trend line of linear regression. The corresponding equations and coefficients of determination (r2) are displayed in the plot area. (JPG 297 kb) [file 11356_2019_4856_MOESM3_ESM.jpg]
